# How words impact on pain

**DOI:** 10.1002/brb3.1377

**Published:** 2019-08-01

**Authors:** Alexander Ritter, Marcel Franz, Wolfgang H. R. Miltner, Thomas Weiss

**Affiliations:** ^1^ Section of Neurological Rehabilitation Hans–Berger Department of Neurology Jena University Hospital Jena Germany; ^2^ Institute of Psychology Friedrich Schiller University of Jena Jena Germany

**Keywords:** embodiment, functional magnetic resonance imaging, pain, priming, semantic processing

## Abstract

**Introduction:**

The wording used before and during painful medical procedures might significantly affect the painfulness and discomfort of the procedures. Two theories might account for these effects: the motivational priming theory (Lang, 1995, *American Psychologist*, **50**, 372) and the theory of neural networks (Hebb, 1949, *The organization of behavior*. New York, NY: Wiley; Pulvermuller, 1999, *Behavioral and Brain Sciences*, **22**, 253; Pulvermüller and Fadiga, 2010, *Nature Reviews Neuroscience*, **11**, 351).

**Methods:**

Using fMRI, we investigated how negative, pain‐related, and neutral words that preceded the application of noxious stimuli as priming stimuli affect the cortical processing and pain ratings of following noxious stimuli.

**Results:**

Here, we show that both theories are applicable: Stronger pain and stronger activation were observed in several brain areas in response to noxious stimuli preceded by both, negative and pain‐related words, respectively, as compared to preceding neutral words, thus supporting motivational priming theory. Furthermore, pain ratings and activation in somatosensory cortices, primary motor cortex, premotor cortex, thalamus, putamen, and precuneus were even stronger for preceding pain‐related than for negative words supporting the theory of neural networks.

**Conclusion:**

Our results explain the influence of wording on pain perception and might have important consequences for clinical work.

## 1 INTRODUCTION

Physically identical noxious stimuli to the skin are not always perceived uniformly painful and unpleasant. Several factors are known to modulate the perception of physically identical noxious stimuli, for example, attention, learning, expectations, emotions, or the social context (Birbaumer, Flor, Lutzenberger, & Elbert, 1995; Flor, 2012; Flor, Birbaumer, & Turk, 1990; Klossika et al., 2006). Recently, it has been shown that words can prime the perception of painful stimuli administered shortly after verbal cues (Richter et al., 2014). Because such priming effects may also exert a persistent impact on the nervous system (Cave & Squire, 1992), wording prior to or during medical procedures is of crucial importance for the perception and discomfort of pain. Patients report more pain and discomfort in response to a medical procedure when the preceding explanation of the procedure addresses the painfulness of the procedure by using pain‐related wording (Dutt‐Gupta, Bown, & Cyna, 2007; Ott, Aust, Nouri, & Promberger, 2012; Wang et al., 2008). This impact of preceding verbal information might be explained by the motivational priming theory (Lang, 1995) positing that negative emotional priming (as with pain‐describing words) might increase arousal and pain emotion, and activate pain memories in response to noxious stimuli. There is evidence that this theory holds true for several types of primes including pictures (Arnold et al., 2008; Kenntner‐Mabiala, Weyers, & Pauli, 2007) and words (Kelly, Lloyd, Nurmikko, & Roberts, 2007; Richter, Eck, Straube, Miltner, & Weiss, 2010). Rhudy and colleagues (Rhudy, McCabe, & Williams, 2007; Rhudy, Williams, McCabe, Russell, & Maynard, 2008), for example, found evidence for the motivational priming theory when individuals saw unpleasant as compared to neutral pictures prior to noxious stimulation. They found increased heart rate, nociceptive flexion reflex, and skin conductance responses to physically identical noxious stimuli when negative as compared to positive pictures were shown. Thus, it may be well assumed that similar consequences might occur when individuals are confronted with pain‐related words.

The theory of neural networks (TNN) (Hebb, 1949) claims that strongly connected cell assemblies will be formed between semantic memory and motor or somatosensory neural networks when neurons of semantic brain areas are frequently activated simultaneously with motor or somatosensory brain areas. For example, studies by Pulvermüller et al. (Pulvermuller, 1999; Pulvermüller & Fadiga, 2010) showed that the presentation of action words such as singing and throwing not only activated semantic memory networks but simultaneously also representations of the mouth or arm in the primary motor cortex indicating that language might be embodied. Based on TNN and the theory of language embodiment, Richter and colleagues observed similar effects to pain‐related words that induced pain sensations although no painful stimuli were presented (Eck, Richter, Straube, Miltner, & Weiss, 2011; Richter et al., 2010, 2014).

**Figure 1:**
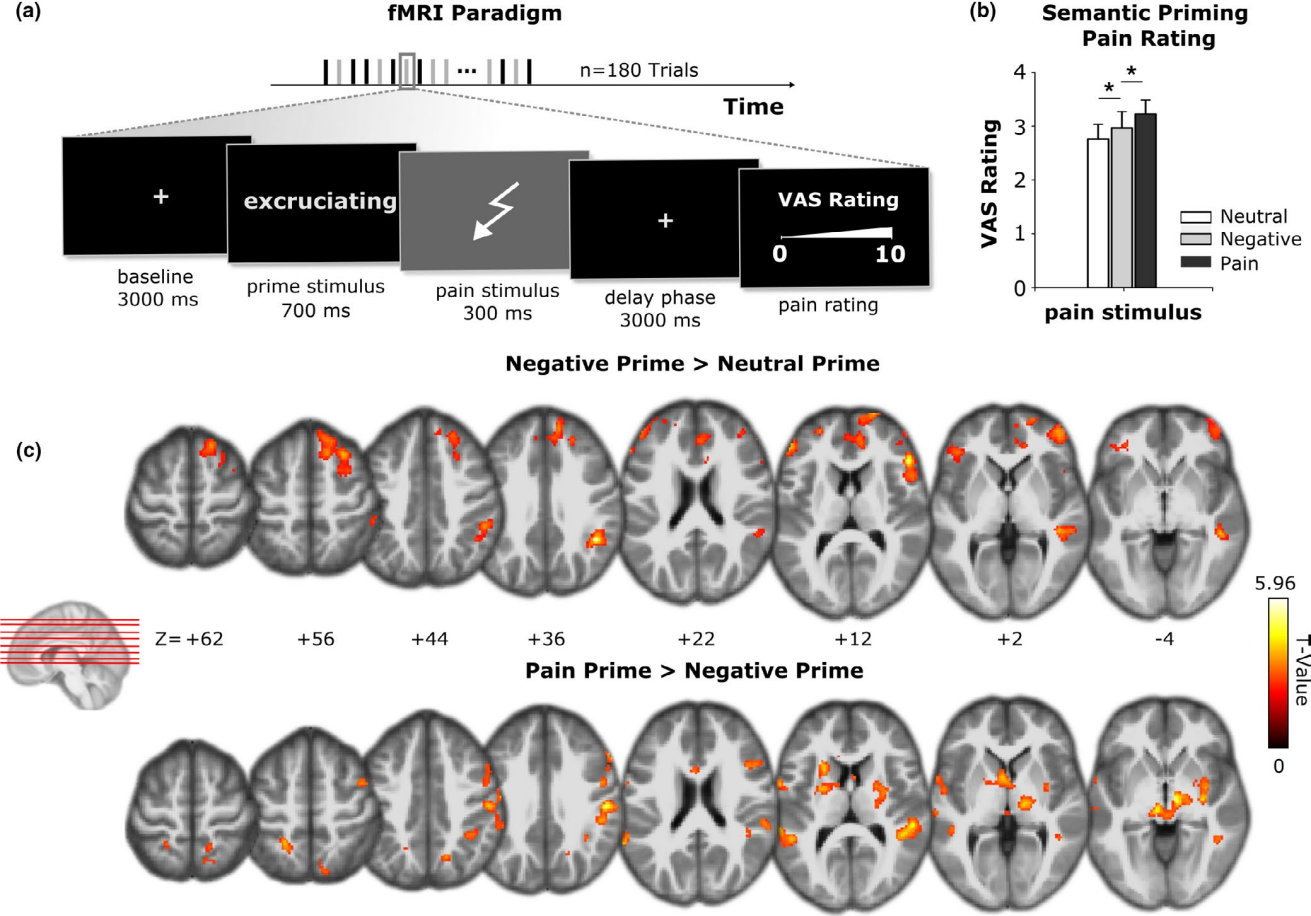
(a) Experimental design. Stimuli were presented in 180 trials. In 90 trials, the words were presented with a subsequent electrical stimulus applied to the tip of the right index finger. The stimulus was followed by a delay phase, and then, the subject was requested to provide a pain rating. In another 90 trials, words were presented without electrical stimulation. (b) Mean pain ratings (±*SE*) for low‐ and high‐intensity electrical stimulation following words with either neutral, negative, or pain‐related words. (c) upper row: comparison of activation to painful stimulation after presentation of negative versus neutral words; lower row: comparison of activation to painful stimulation preceded by pain‐related versus by negative words. The summary statistic images were thresholded at uncorrected *p* = .01 with FWE correction at cluster level, *p *= .05, based on random field theory

Thus, specific predictions are possible for the priming effects evoked by negative versus pain‐related words compared with neutral words: Elevated pain ratings and stronger cortical activation should be observed when pain‐related and negative words compared with neutral words would be used as primes preceding or during painful stimulation. Additionally, TNN would predict that pain‐related words also directly might activate the neural networks that constitute the experience of pain. To test whether the processing of noxious input in the brain might become differentially modulated by different types of verbal primes, we conducted a functional magnetic resonance imaging (fMRI) experiment where subjects were exposed to painful electrical stimuli that were preceded either by pain‐related, non‐pain‐related negative, or neutral words (Figure 1a).

## 2 MATERIALS AND METHODS

### 2.1 Study subjects

Seventeen healthy participants (11 females and 6 males, mean age ± *SD* 23.3 ± 3.1 years, range: 20–31 years) took part in the experiment. We recruited subjects from the undergraduate psychology program of the Friedrich Schiller University of Jena. All participants gave written informed consent and were free to terminate their participation in the experiment at any time without negative consequences. The Ethics Committee of the University of Jena approved the experiment.

### 2.2 Stimuli

Visually presented verbal primes were composed of pain‐related, non‐pain‐related negative, and neutral words. Words were matched with respect to word length, number of syllables, and frequency of words used in German language (according to COSMAS II http://www.ids-mannheim.de/cosmas2/). Word stimuli were selected according to Richter et al. (2010). Pain‐related words were collected from pain questionnaires (e.g., McGill Pain Questionnaire) whereas words of other categories were obtained according to previous studies on word perception of our group (Dillmann, Miltner, & Weiss, 2000; Weiss, Miltner, & Dillmann, 2003). Overall, 156 emotional adjectives were rated for valence, arousal, and pain relatedness on three rating scales ranging from 0 to 10, for example, 0 = not pain‐related and 10 = strongly pain‐related. Words producing significant differences between male and female participants on these scales were excluded. Pain relevance ratings for neutral words were 0.31 ± 0.17, for negative words 1.05 ± 0.25, and for pain‐related words 3.14 ± 0.38. Pain‐related words and non‐pain‐related negative words were matched for arousal and valence. In the current experiment, each word was shown for 700 ms. Electrical stimuli consisted of biphasic constant current square wave pulses (DS5; Digitimer) with a duration of 300 ms each. They were delivered by a concentric surface electrode with a central cathode (diameter: 0.5 mm) and an external anode ring (diameter: 5 mm; K2 stimulation electrode; Walter Graphtek Corporation), designed to increase the current field density and to depolarize nociceptive fibers within the epidermis predominantly.

### 2.3 Experimental procedure

The experiment took place in a quiet, temperature‐controlled (21–23°C) room. The stimulation procedure was controlled by Presentation^®^ software (Version 18.3; Neurobehavioral Systems, Inc., www.neurobs.com). Before scanning, subjects underwent a thresholding to determine the intensity of electrical stimuli using the method of limits. They were asked to increase the electrical stimulus intensity stepwise by pressing an up and down button on a computer keyboard until pain rating reached 3 for “low pain intensity” and 5 for “high pain intensity” on a VAS (visual analogue rating scale) ranging from 0 (no pain) to 10 (strongest pain imaginable). Following determination of low pain intensity, the thresholding was repeated for high pain intensity. Both thresholds were established for participant's right hand.

Then, while in the scanner, participants were familiarized with the experimental procedure by presenting five trials with either a high or low electrical stimulus intensity or without electrical stimulation. The prime words were projected via a video beamer onto a screen mounted on the head coil of the scanner above participants' eyes. Participants were asked to focus their attention on the screen. Prime stimuli were presented in 180 trials. For one half of trials, primes were presented with a succeeding electrical stimulus (45 belonging to each intensity) whereas for the other half of trials only word primes were presented without succeeding electrical stimuli. These 90 trials without subsequent electrical stimulation as well as the two different intensities in trials with electrical stimulation were presented to control effects of habituation and expectation. All trials were presented in pseudorandom order with the restriction that the same word category (neutral, negative, or pain‐related) was not presented directly on each other. Trails with electrical stimulation were followed by a delay during which a fixation cross was presented for three seconds followed by an interval during which subjects were requested to rate the stimulus intensity on the VAS scale. VAS scale was the same as in the thresholding procedure (see above). Subjects responded by pressing a button fixed below their left hand. After the pain rating, a fixation cross was presented at the monitor screen for eight seconds. The whole fMRI procedure took about 30 min depending on the time the subjects required for their pain ratings.

### 2.4 Analysis of behavioral data

To test whether VAS responses to painful electrical stimulation differ between low and high stimulus intensity as well as between the Word Categories a linear mixed‐effects model (LMEM) using Stimulus Intensity and Word Category as fixed effects. The parameters of fixed effects such as *β*_0_ (intercept or baseline level) and *β*_1_ (slope or treatment effect) are assumed to be constant across experiments (Barr, Levy, Scheepers, & Tily, 2013). To account for between‐individual variation in VAS response, subject ID was included as random effect: specifically, we added by‐subject random intercepts and random slopes to the LMEM. By including random intercepts, that is, deviations from the fixed‐effect* β*_0_, we allow the intercept term to vary across subjects (*u*_0S_), which accounts for the fact that different subjects are likely to have different overall VAS ratings. Likewise, including random slopes (*u*_1S_) we allow the fixed‐effect *β*_1_ to vary across subjects, thereby assuming that different subjects may respond differently to the experimental factors. The covariance structure of random effects was specified as variance component structure, that is, a diagonal matrix with unrestricted main diagonal entries enabling a different variance component for each random effect and off‐diagonal entries set to zero, that is, assuming no correlation between random effects. LMEM was fitted using restricted maximum likelihood estimation. As the interaction between Word Category and Stimulus Intensity was significant, pairwise comparisons were conducted. To counteract the problem of multiple comparisons, significance level was adjusted with Bonferroni correction for the six estimates. All statistical calculations were carried out using IBM SPSS Statistics 24 (SPSS Inc.). We considered values of *p* < .05 to be statistically significant.

### 2.5 Imaging

In a 3 Tesla magnetic resonance scanner (Tim Trio, Siemens, Medical Systems, Erlangen, Germany), 35 slices were collected using a T2* weighted echo‐planar sequence (time to echo [TE] = 30 ms, flip angle = 90°, matrix = 64 × 64, field of view [FOV] = 192 mm, scan repeat interval [TR] = 2.08 s, thickness = 3 mm, 0.51 mm gap, in‐plane resolution = 3 × 3 mm) parallel to the intercommissural plane (AC‐PC‐plane). Additionally, a high‐resolution T1‐weighted anatomical volume was recorded (192 slices, TE = 5 ms, matrix = 256 × 256 mm, resolution = 1 × 1 × 1 mm). Image processing and statistical analysis of fMRI data were performed using SPM12 (www.fil.ion.ucl.ac.uk/spm/). Preprocessing included slice timing and realignment to the first volume. Because painful electrical stimuli often evoke involuntary movements that are correlated with stimulus onset, we corrected for the interaction of head motion and the inhomogeneities of the magnetic field (susceptibility movement interaction) using the unwarping procedure of SPM12. The maximum amount of head motion did not exceed 1.8 mm in any of the participants. The anatomical volume was coregistered with the mean echo‐planar image. Both structural and functional volumes were normalized to standard Montreal Neurological Institute space using the transformation matrix obtained after coregistration. Functional images were smoothed with an 8 mm Gaussian kernel with full‐width at half‐maximum (FWHM). Data analysis was performed using the general linear model (GLM). On the subject level, the model contained three by three regressors that coded for the nine experimental conditions. For each valence of the words (negative, neutral, pain‐related), three regressors were determined: presentation of words alone, presentation of words with subsequent low‐intensity painful stimulation, and presentation of words with subsequent high‐intensity painful stimulation. An additional regressor‐of‐no‐interest coded the manual rating period after stimulus presentation. Each boxcar stimulus function was convolved with a canonical hemodynamic response function, and data were high‐pass filtered with a cutoff period of 128 s. The effects of interest were tested using linear contrasts of the parameter estimates for the particular regressor, resulting in a *t*‐statistic for each voxel. In a next step, separate contrast images representing the difference between pain‐related versus negative words, negative versus neutral words, and pain‐related versus neutral words preceding high‐intensity painful stimulation were generated for each participant, which were subsequently included to a second GLM. At the group level, a random‐effects approach was used (Friston, Holmes, & Worsley, 1999), treating inter‐subject variability as a random factor. The summary statistic images were thresholded at uncorrected *p* = .01 with FWE correction at cluster level, *p* = .05, based on random field theory (Lieberman & Cunningham, 2009). This resulted in a minimal cluster extent of 165 contiguous voxels for the comparison between pain‐related and negative words, 167 contiguous voxels for the comparison between negative and neutral words, and 200 contiguous voxels for the comparison between pain‐related and neutral words.

## 3 RESULTS

Different from pain ratings obtained in the thresholding procedure of 3 and 5 on VAS for low and high stimulus intensity, respectively, VAS ratings during scanning were generally lower (high stimulus intensity, negative: *M* = 2.97, *SD* = 1.24, neutral: *M* = 2.76, *SD* = 1.13, pain‐related: *M* = 3.22, *SD* = 1.06; low stimulus intensity, negative: *M* = 1.21, *SD* = 0.74, neutral: *M* = 1.06, *SD* = 0.71, pain‐related: *M* = 1.05, *SD* = 0.70). Obviously, circumstances in the scanner biased pain perception. This might have been due to distraction from the electrical stimulation and general higher during scanning.

Using a linear mixed‐effect model (LMEM), analysis of pain ratings revealed significant fixed effects for Stimulus Intensity (low, high), *F*_1,16_ = 43.66, *p* < .001 and for Word Category (negative, neutral, pain‐related), *F*_2,1,492_ = 6.956, *p* = .001 as well as a significant interaction between Stimulus Intensity and Word Category *F*_2,1,492_ = 6.560, *p* = .001. As pairwise comparisons revealed, painful electrical stimuli with high intensity were rated more painful when pain‐related words or words with negative valence were presented before painful stimulation as compared to neutral words (negative vs. neutral words: difference of means = 0.211, 95% CI for difference = 0.012–0.411, *p* = .017; pain‐related vs. neutral words: difference of means = 0.395, 95% CI for difference = 0.195–0.594, *p* < .001). However, according to the TNN concept, physically identical electrical stimuli were rated more painful when preceded by a pain‐related than a negative non‐pain‐related word (difference of means = 0.183, 95% CI for difference = −0.17 to 0.383, *p* = .042, see Figure 1b). Low stimulus intensity trials did not differ significantly between word categories. As a consequence, the priming effect for the words most likely depends on sufficiently strong pain intensity. Taken together, both, motivational priming theory and TNN, have proven applicable to explain the results.

In accordance with these behavioral observations, fMRI revealed brain activation patterns predicted by both theories. Noxious stimuli preceded by pain‐related or negative words induced stronger brain activation than neutral words in anterior cingulate cortex (ACC) and dorsolateral prefrontal cortex (DLPFC), among others (Figure 1c, Table S1). Again, painful stimuli preceded by pain‐related words evoked stronger activations in a number of brain regions, including DLPFC, ACC, and precuneus (Table S2) as compared to painful stimuli preceded by neutral words. Painful stimuli following pain‐related words indicated stronger activation in secondary somatosensory cortex (SII), primary motor cortex (MI), putamen, nucleus caudatus, thalamus, periaqueductal gray, and precuneus (Table 1, Figure 1c) than painful stimuli following negative words.

**Table 1:** Clusters of activation to identical painful electrical stimuli preceded by pain‐related versus negative words

Region label	Extent	*t*‐Value	*x*	*y*	*z*	Brodmann area
L/R Putamen	469	5.967	4	6	16	49
L Inferior occipital Gyrus, Fusiform Gyrus, Cerebellum (VI)	381	5.685	−36	−64	−16	19/37
L/R Thalamus, Periaqueductal Gray, Hippocampus	810	5.241	14	−18	0	50/54
R Postcentral Gyrus, Supramarginal Gyrus	419	5.184	52	−22	36	7/40
L Inferior/Middle/Superior Temporal Gyrus, Supramarginal Gyrus	411	5.157	−60	−36	10	21/22/40
R Inferior/Middle/Superior Temporal Gyrus, Supramarginal Gyrus	500	4.8	54	−34	14	21/22/40
R Inferior Frontal Gyrus, Precentral Gyrus	556	3.969	56	22	32	44/6/9
L Caudate Nucleus	235	3.903	−4	4	2	48
L Postcentral Gyrus, Inferior Parietal Lobule	168	3.797	−22	−48	54	7
L Inferior Frontal Gyrus, Rolandic Operculum, Postcentral Gyrus	209	3.687	−60	2	12	44/6/7
R Precuneus, Angular Gyrus, Cuneus, Postcentral Gyrus	294	3.625	18	−60	42	39/7

Note

Listed are clusters of activation with a voxel threshold of *p* < .01 and a cluster threshold of *p* < .01 (165 contiguous voxels). MNI coordinates are provided for the maxima of the respective cluster. The corresponding neuroanatomical regions, the Brodmann areas, and the laterality (L, left; R, right) are described.

## 4 DISCUSSION

### 4.1 Theory of neural networks (TNN) and motivational priming theory

Behavioral and fMRI data of the present experiment revealed significantly different effects of preceding verbal information on painful stimuli. Pain‐related and negative words significantly enhanced the pain intensity of painful stimuli compared with neutral words in response to physically identical painful stimuli. This is in accordance with the motivational priming theory. Moreover, as predicted by TNN, pain‐related words additionally increased the pain intensity in comparison to negative words without pain relatedness and stronger brain activations were found in the somatosensory cortex (SII, somatosensory association cortex [SAC]) and motor cortex (MI, premotor cortex). With respect to TNN, activation in body‐related brain areas as SII and SAC further indicates that pain‐related verbal cues do not necessarily have to be body‐related. Similar effects of embodiment have been reported previously during the processing of verbs belonging to activities of leg versus arm versus face evoking activities in the sensorimotor areas of the respective representation (Pulvermuller, 2013). As the putamen also shows somatotopic organization when painful stimuli are applied (Bingel, Glascher, Weiller, & Buchel, 2004; Bingel, Lorenz, et al., 2004), the stronger activation in this structure suggests that even midbrain structures may constitute the embodied network representation of pain‐related words. Alternatively, the stronger activation of the putamen may have contributed to the stronger pain ratings because of its role in encoding of stimulus intensity (Chudler, 1998; Chudler & Dong, 1995). Stronger activations were also found in the face area of MI and in premotor cortex. Thus, pain‐related words in comparison with non‐pain‐related negative words also activated brain areas relevant for motor activities such as pain‐related facial expressions and withdrawal responses (Moseley, Carota, Hauk, Mohr, & Pulvermuller, 2012). Importantly, pain‐related but not negative words induced activity in motor areas pointing to pain‐related semantics of this word category that got embodied. Taken together, pain‐related words—in contrast to negative words—presumably activated body‐ and action‐related areas of somatosensory and motor areas of the brain.

### 4.2 Possible role of expectations

One might argue that our results could be explained, at least in part, by expectations. For example, minimizing positive expectancies about analgesic treatments was able to diminish pain relief (Kong et al., 2009). This effect was also apparent in corresponding fMRI data, that is, somatotopically organized brain areas as MI and SI showed modulated activity for the body regions which were manipulated with expectations about pain. Other than in the current study, expectations were generated explicitly by the examiners through manipulating movies. Thus, cognitive influences on pain perception (Wiech, 2016) are evident. Importantly, in the current study word stimuli were not associated with suggestions of any kind and, therefore, influenced subsequent painful stimuli with their mere signification. Applying the Bayesian coding hypothesis (Knill & Pouget, 2004) to our experiment, one might argue that the participants were continuously generating and updating information about the words and the forthcoming electrical stimulation over the course of the experiment. In order to infer the stimulation strength, that is, the painfulness of the electrical pulses and to gather control for the upcoming pain stimulus, predictions might have been formed for the different words as priming stimuli. As words of all categories were followed by either no, weak or strong pain stimulation with equal likelihood, such predictions proofed incorrect most of the time. As a consequence, we infer that concrete and correct expectations about intensity of the forthcoming stimulus were unlikely. Therefore, expectations are less applicable to explain the differences in pain rating and cortical activity. Therefore, differences in pain perception shown between word categories most likely result from priming effects as predicted by the motivational priming theory and TNN. In the current paradigm, words were selected with respect to arousal, valence, and pain relatedness. Word categories were not consequently separated considering the aspect of body relatedness as some words directly portend to body parts whereas others do not. To further disentangle the relevance of body‐ and non‐body‐related verbal cues for the expression of pain, it would be reasonable to compare both types of words directly. That has not been possible in the current study due to different word counts in the respective sub‐categories. Modeling body relatedness as a factor, one could directly examine the impact of this aspect on neural processing. For now, the semantically embodied concepts of words are based on the fact that the emotional relevance of the adjectives clearly suggests a specific physical reaction. In that sense, pain‐specific verbal content per definition implies a bodily component. Thus, the specific additional priming effect of pain‐related words is most likely due to their body‐related pain specificity and the concomitant activation in the neural network.

A more general limitation is the low number of participants. Low participant counts are generally capable for effects in fMRI but possibly do not depict the behavioral effects to their full extent. In this context, we find another drawback in our sample concerning gender balance. Most of our participants were women which is a consequence of the gender distribution in life sciences at University of Jena. As it has previously been found that gender is crucial for pain experience (Greenspan et al., 2007), it should be investigated in future studies.

Considering our results, we suggest that both, motivational priming theory and TNN, account for the stronger pain ratings when painful stimuli were preceded by pain‐related verbal cues. We show that pain‐related—as compared to neutral or to non‐pain‐related negative—adjectives increase the pain intensity and concomitantly induce brain activation in specific brain areas in response to noxious stimuli. Accordingly, our results offer a pain‐specific extension for the TNN and the theory of embodiment theory of language. Of practical relevance, enhanced pain and discomfort during a medical procedure may, therefore, be a consequence of verbally activated embodied pain‐related concepts. Consequently, it would be reasonable to concentrate on less pain‐accented expressions within painful medical procedures and to restrain on formulations of well‐being wherever applicable for the course of the operation.

## CONFLICT OF INTEREST

None declared.

## Data Availability

The datasets generated during and/or analyzed during the current study are available from the corresponding author on reasonable request.

## References

[brb31377-bib-0001] Arnold, B. S. , Alpers, G. W. , Suss, H. , Friedel, E. , Kosmutzky, G. , Geier, A. , & Pauli, P. (2008). Affective pain modulation in fibromyalgia, somatoform pain disorder, back pain, and healthy controls. European Journal of Pain, 12, 329–338. 10.1016/j.ejpain.2007.06.007 17723312

[brb31377-bib-0002] Barr, D. J. , Levy, R. , Scheepers, C. , & Tily, H. J. (2013). Random effects structure for confirmatory hypothesis testing: Keep it maximal. Journal of Memory and Language, 68, 255–278. 10.1016/j.jml.2012.11.001 PMC3881361 24403724

[brb31377-bib-0003] Bingel, U. , Glascher, J. , Weiller, C. , & Buchel, C. (2004). Somatotopic representation of nociceptive information in the putamen: An event‐related fMRI study. Cerebral Cortex, 14, 1340–1345. 10.1093/cercor/bhh094 15217895

[brb31377-bib-0004] Bingel, U. , Lorenz, J. , Glauche, V. , Knab, R. , Glascher, J. , Weiller, C. , & Buchel, C. (2004). Somatotopic organization of human somatosensory cortices for pain: A single trial fMRI study. NeuroImage, 23, 224–232. 10.1016/j.neuroimage.2004.05.021 15325369

[brb31377-bib-0005] Birbaumer, N. , Flor, H. , Lutzenberger, W. , & Elbert, T. (1995). The corticalization of chronic pain In Bromm B., Desmedt J. E. (Eds.), Pain and the brain: From nociception to cognition (pp. 331–343). New York, NY: Raven Press.

[brb31377-bib-0006] Cave, C. B. , & Squire, L. R. (1992). Intact and long‐lasting repetition priming in amnesia. Journal of Experimental Psychology. Learning, Memory, and Cognition, 18, 509–520. 10.1037/0278-7393.18.3.509 1534352

[brb31377-bib-0007] Chudler, E. H. (1998). Response properties of neurons in the caudate‐putamen and globus pallidus to noxious and non‐noxious thermal stimulation in anesthetized rats. Brain Research, 812, 283–288. 10.1016/S0006-8993(98)00971-8 9813370

[brb31377-bib-0008] Chudler, E. H. , & Dong, W. K. (1995). The role of the basal ganglia in nociception and pain. Pain, 60, 3–38. 10.1016/0304-3959(94)00172-B 7715939

[brb31377-bib-0009] Dillmann, J. , Miltner, W. H. R. , & Weiss, T. (2000). The influence of semantic priming on event‐related potentials to painful laser‐heat stimuli in humans. Neuroscience Letters, 284, 53–56. 10.1016/S0304-3940(00)00957-5 10771160

[brb31377-bib-0010] Dutt‐Gupta, J. , Bown, T. , & Cyna, A. M. (2007). Effect of communication on pain during intravenous cannulation: A randomized controlled trial. British Journal of Anaesthesia, 99, 871–875. 10.1093/bja/aem308 17977860

[brb31377-bib-0011] Eck, J. , Richter, M. , Straube, T. , Miltner, W. H. R. , & Weiss, T. (2011). Affective brain regions are activated during the processing of pain‐related words in migraine patients. Pain, 152, 1104–1113. 10.1016/j.pain.2011.01.026 21377797

[brb31377-bib-0012] Flor, H. (2012). New developments in the understanding and management of persistent pain. Current Opinion in Psychiatry, 25, 109–113. 10.1097/YCO.0b013e3283503510 22227632

[brb31377-bib-0013] Flor, H. , Birbaumer, N. , & Turk, D. C. (1990). The Psychobiology of chronic pain. Advances in Behaviour Research and Therapy, 12, 47–84. 10.1016/0146-6402(90)90007-D

[brb31377-bib-0014] Friston, K. J. , Holmes, A. P. , & Worsley, K. J. (1999). How many subjects constitute a study? NeuroImage, 10, 1–5. 10.1006/nimg.1999.0439 10385576

[brb31377-bib-0015] Greenspan, J. D. , Craft, R. M. , LeResche, L. , Arendt‐Nielsen, L. , Berkley, K. J. , Fillingim, R. B. , … Pain, S.I.G.o.t.I (2007). Studying sex and gender differences in pain and analgesia: A consensus report. Pain, 132(Suppl 1), S26–S45. 10.1016/j.pain.2007.10.014 17964077 PMC2823483

[brb31377-bib-0016] Hebb, D. O. (1949). The organization of behavior. New York, NY: Wiley.

[brb31377-bib-0017] Kelly, S. , Lloyd, D. , Nurmikko, T. , & Roberts, N. (2007). Retrieving autobiographical memories of painful events activates the anterior cingulate cortex and inferior frontal gyrus. Journal of Pain, 8, 307–314. 10.1016/j.jpain.2006.08.010 17188577

[brb31377-bib-0018] Kenntner‐Mabiala, R. , Weyers, P. , & Pauli, P. (2007). Independent effects of emotion and attention on sensory and affective pain perception. Cognition Emotion, 21, 1615–1629. 10.1080/02699930701252249

[brb31377-bib-0019] Klossika, I. , Flor, H. , Kamping, S. , Bleichhardt, G. , Trautmann, N. , Treede, R. D. , … Schmahl, C. (2006). Emotional modulation of pain: A clinical perspective. Pain, 124, 264–268. 10.1016/j.pain.2006.08.007 16934927

[brb31377-bib-0020] Knill, D. C. , & Pouget, A. (2004). The Bayesian brain: The role of uncertainty in neural coding and computation. Trends in Neurosciences, 27, 712–719. 10.1016/j.tins.2004.10.007 15541511

[brb31377-bib-0021] Kong, J. , Kaptchuk, T. J. , Polich, G. , Kirsch, I. , Vangel, M. , Zyloney, C. , … Gollub, R. L. (2009). An fMRI study on the interaction and dissociation between expectation of pain relief and acupuncture treatment. NeuroImage, 47, 1066–1076. 10.1016/j.neuroimage.2009.05.087 19501656 PMC2742363

[brb31377-bib-0022] Lang, P. J. (1995). The emotion probe: Studies of motivation and attention. American Psychologist, 50, 372–385. 10.1037/0003-066X.50.5.372 7762889

[brb31377-bib-0023] Lieberman, M. D. , & Cunningham, W. A. (2009). Type I and Type II error concerns in fMRI research: Re‐balancing the scale. Social Cognitive and Affective Neuroscience, 4, 423–428. 10.1093/scan/nsp052 20035017 PMC2799956

[brb31377-bib-0024] Moseley, R. , Carota, F. , Hauk, O. , Mohr, B. , & Pulvermuller, F. (2012). A role for the motor system in binding abstract emotional meaning. Cerebral Cortex, 22, 1634–1647. 10.1093/cercor/bhr238 21914634 PMC3377965

[brb31377-bib-0025] Ott, J. , Aust, S. , Nouri, K. , & Promberger, R. (2012). An everyday phrase may harm your patients the influence of negative words on pain during venous blood sampling. Clinical Journal of Pain, 28, 324–328. 10.1097/AJP.0b013e3182321cc3 22001664

[brb31377-bib-0026] Pulvermuller, F. (1999). Words in the brain's language. Behavioral and Brain Sciences, 22, 253–279, 327–336. 11301524

[brb31377-bib-0027] Pulvermuller, F. (2013). How neurons make meaning: Brain mechanisms for embodied and abstract‐symbolic semantics. Trends in Cognitive Sciences, 17, 458–470. 10.1016/j.tics.2013.06.004 23932069

[brb31377-bib-0028] Pulvermüller, F. , & Fadiga, L. (2010). Active perception: Sensorimotor circuits as a cortical basis for language. Nature Reviews Neuroscience, 11, 351–360. 10.1038/nrn2811 20383203

[brb31377-bib-0029] Rhudy, J. L. , McCabe, K. M. , & Williams, A. E. (2007). Affective modulation of autonomic reactions to noxious stimulation. International Journal of Psychophysiology, 63, 105–109. 10.1016/j.ijpsycho.2006.09.001 17049399

[brb31377-bib-0030] Rhudy, J. L. , Williams, A. E. , McCabe, K. M. , Russell, J. L. , & Maynard, L. J. (2008). Emotional control of nociceptive reactions (ECON): Do affective valence and arousal play a role? Pain, 136, 250–261. 10.1016/j.pain.2007.06.031 17703886

[brb31377-bib-0031] Richter, M. , Eck, J. , Straube, T. , Miltner, W. H. R. , & Weiss, T. (2010). Do words hurt? Brain activation during the processing of pain‐related words. Pain, 148, 198–205. 10.1016/j.pain.2009.08.009 19846255

[brb31377-bib-0032] Richter, M. , Schroeter, C. , Puensch, T. , Straube, T. , Hecht, H. , Ritter, A. , … Weiss, T. (2014). Pain‐related and negative semantic priming enhances perceived pain intensity. Pain Research and Management, 19, 69–74. 10.1155/2014/425321 24716197 PMC4028655

[brb31377-bib-0033] Wang, F. , Shen, X. , Xu, S. , Liu, Y. , Ma, L. , Zhao, Q. , … Li, X. (2008). Negative words on surgical wards result in therapeutic failure of patient‐controlled analgesia and further release of cortisol after abdominal surgeries. Minerva Anestesiologica, 74, 353–365. 18612266

[brb31377-bib-0034] Weiss, T. , Miltner, W. H. R. , & Dillmann, J. (2003). The influence of semantic priming on event‐related potentials to painful laser‐heat stimuli in migraine patients. Neuroscience Letters, 340, 135–138. 10.1016/S0304-3940(03)00103-4 12668255

[brb31377-bib-0035] Wiech, K. (2016). Deconstructing the sensation of pain: The influence of cognitive processes on pain perception. Science, 354, 584–587. 10.1126/science.aaf8934 27811269

