# Supplementary material for: How words impact on pain
Source: Brain Behav. 2019 Aug 1;9(9):e01377. doi: 10.1002/brb3.1377 (PMC6749494; doi:10.1002/brb3.1377)
Supplement: Supplementary file 1 [file BRB3-9-e01377-s001.docx]

Supplementary Table 1. Clusters of activation to identical painful electrical stimuli preceded by negative vs. neutral words

| Region Label | Extent | t-value | x | y | z | Brodmann Area |
| --- | --- | --- | --- | --- | --- | --- |
|  |  |  |  |  |  |  |
| R SupraMarginal Gyrus, Angular Gyrus | 525 | 6,854 | 44 | -48 | 36 | 39/40 |
| R Inferior Frontal Gyrus | 437 | 6,318 | 50 | 26 | 14 | 44/45 |
| L Inferior/ Middle Frontal Gyrus, Middle Orbital Gyrus | 626 | 6,126 | -56 | 30 | 18 | 46/10 |
| R Superior Frontal Gyrus /ACC/Superior Medial Gyrus | 2250 | 5,465 | 18 | 66 | 10 | 8/9/32 |
| R Middle Temporal Gyrus | 269 | 5,110 | 48 | -36 | -6 | 21 |
| L Cerebelum (VII, Crus 1) | 308 | 4,951 | -10 | -76 | -30 |  |
| R Middle Orbital Gyrus, Middle Frontal Gyrus | 413 | 4,270 | 42 | 50 | 0 | 10/46 |

Listed are clusters of activation with a voxel threshold of *P*<0.01 and a cluster threshold of *P*<0.01 (167 contiguous voxels). MNI coordinates are provided for the maxima of the respective cluster. The corresponding neuroanatomical regions, the Brodmann areas, and the laterality (L, left; R, right) are described.

Supplementary Table 2. Clusters of activation to identical painful electrical stimuli preceded by pain-related vs. neutral words

| Region Label | Extent | t-value | x | y | z | Brodmann Area |
| --- | --- | --- | --- | --- | --- | --- |
|  |  |  |  |  |  |  |
| R Thalamus | 7018 | 8.643 | 16 | -18 | 2 | 50 |
| Cerebellar Vermis (3) | 7018 | 7.043 | 2 | -48 | -14 |  |
| R Superior temporal Gyrus | 7018 | 6.017 | 42 | -32 | 2 |  |
| R IFG (p. Triangularis) | 5640 | 6.28 | 48 | 30 | 12 | 46 |
| R Posterior-Medial Frontal | 5640 | 4.719 | 10 | 14 | 60 | 48 |
| R Middle Orbital Gyrus | 5640 | 4.465 | 44 | 54 | 4 | 10 |
| L Superiotr temporal Gyrus | 3604 | 5.302 | -42 | -16 | -10 |  |
| L IFG (p. Triangularis) | 3604 | 5.248 | -56 | 12 | 10 | 44 |
| R Inferior Parietal Lobule | 4420 | 5.259 | 36 | -48 | 38 | 39 |
| R Precuneus | 4420 | 5.15 | 12 | -70 | 64 | 7 |
| R Superior Occipital Gyrus | 4420 | 4.775 | 24 | -74 | 48 | 7 |
| L Superior temporal Gyrus | 1135 | 4.554 | -44 | -46 | 26 |  |
| L Middle Temporal Gyrus | 1135 | 4.168 | -54 | -38 | 8 | 22 |
| L Inferior Parietal Lobule | 1135 | 3.861 | -52 | -46 | 46 | 39 |
| L Middle Frontal Gyrus | 244 | 3.631 | -24 | 40 | 34 | 9 |
| R ACC | 244 | 3.189 | 10 | 40 | 16 | 32 |
| L Superior Medial Gyrus | 244 | 2.99 | -14 | 20 | 24 |  |
| R IFG (p. Triangularis) | 5640 | 2.661 | 42 | 20 | 10 | 45 |
| R Superior Frontal Gyrus | 5640 | 2.917 | 16 | 14 | 64 |  |

Listed are clusters of activation with a voxel threshold of *P*<0.01 and a cluster threshold of *P*<0.01 (200 contiguous voxels). MNI coordinates are provided for the maxima of the respective cluster. The corresponding neuroanatomical regions, the Brodmann areas, and the laterality (L, left; R, right) are described.
